# Genetic Diversity and Population Genetics of Mosquitoes (Diptera: Culicidae: *Culex* spp.) from the Sonoran Desert of North America

**DOI:** 10.1155/2013/724609

**Published:** 2013-11-03

**Authors:** Edward Pfeiler, Carlos A. Flores-López, Jesús Gerardo Mada-Vélez, Juan Escalante-Verdugo, Therese A. Markow

**Affiliations:** ^1^Centro de Investigación en Alimentación y Desarrollo, A.C., Unidad Guaymas, Apartado Postal 284, 85480 Guaymas, SON, Mexico; ^2^Department of Biology, University of Maryland, College Park, MD 20742, USA; ^3^Instituto de Seguridad y Servicios Sociales de Los Trabajadores del Estado de Sonora (ISSSTESON), 83000 Hermosillo, SON, Mexico; ^4^Laboratorio Estatal de Salud Publica, Zona Edificios Federales, Col. Las Quintas, 83260 Hermosillo, SON, Mexico; ^5^Division of Biological Sciences, University of California, San Diego, La Jolla, CA 92093, USA; ^6^Laboratorio Nacional de Genómica de Biodiversidad-CINVESTAV, 36821 Irapuato, GTO, Mexico

## Abstract

The population genetics and phylogenetic relationships of *Culex* mosquitoes inhabiting the Sonoran Desert region of North America were studied using mitochondrial DNA and microsatellite molecular markers. Phylogenetic analyses of mitochondrial cytochrome *c* oxidase subunit I (COI) from mosquitoes collected over a wide geographic area, including the Baja California peninsula, and mainland localities in southern Arizona, USA and Sonora, Mexico, showed several well-supported partitions corresponding to *Cx. quinquefasciatus, Cx. tarsalis,* and two unidentified species, *Culex* sp. 1 and sp. 2. *Culex quinquefasciatus* was found at all localities and was the most abundant species collected. *Culex tarsalis* was collected only at Tucson, Arizona and Guaymas, Sonora. The two unidentified species of *Culex* were most abundant at Navojoa in southern Sonora. Haplotype and nucleotide diversities in the COI gene segment were substantially lower in *Cx. quinquefasciatus* compared with the other three species. Analysis of molecular variance revealed little structure among seven populations of *Cx. quinquefasciatus*, whereas significant structure was found between the two populations of *Cx. tarsalis*. Evidence for an historical population expansion beginning in the Pleistocene was found for *Cx. tarsalis*. Possible explanations for the large differences in genetic diversity between *Cx. quinquefasciatus* and the other species of *Culex* are presented.

## 1. Introduction

The population structure, dispersal capabilities, and systematics of mosquitoes in the genus *Culex* (Culicidae: Culicinae: Culicini) from the Sonoran Desert of North America are poorly known. Several species reported from this region, including *Cx. quinquefasciatus* Say, a member of the *Cx. pipiens* Linnaeus complex, and *Cx. tarsalis* Coquillett, are important vectors of the West Nile and St. Louis encephalitis viruses that infect humans. Although presently not as serious of a health problem in Mexico as the dengue fever virus vectored by the introduced *Aedes aegypti* (Linnaeus), a single mortality from West Nile viral infection recorded in 2009 in the northern city of Monterrey, Nuevo León [1], and an infection reported from southern Sonora in which the patient later recovered [2], raises concern that there is a potential for this disease to emerge in northern Mexico and that it should be monitored more closely by health officials. Owing to the lack of a vaccine for the West Nile virus, vector control is the only tool presently available to combat this disease. Efficient monitoring of vector control measures, and inferring sources of reinvasion, depends on an understanding of the dispersal capability and genetic diversity of the mosquitoes, as well as on accurate taxonomic identifications. The genus *Culex* contains 768 described species, many of which (198) are grouped in the subgenus *Culex*, and important gaps still exist in our knowledge of their taxonomy and relationships [3, 4]. 

 In the *Cx. pipiens* complex, the species-level taxa present in the New World are *Cx. pipiens *and *Cx. quinquefasciatus* which show an extensive zone of hybridization at mid latitudes in the USA [5–7]. Some authors, however, place *Cx. quinquefasciatus* as a subspecies of *Cx. pipiens* [8]. Here they are treated as separate species. *Culex quinquefasciatus* is widely distributed, found in southern USA, Mexico, Central America, and most of South America [5]. 

*Culex quinquefasciatus* and *Cx. tarsalis* have markedly different histories in the Sonoran Desert, as well as elsewhere in the New World, and a comparison of their population genetics is predicted to reveal patterns reflecting these differences. *Culex tarsalis* is native to North America, whereas both *Cx. pipiens* and *Cx. quinquefasciatus* are thought to have evolved in Africa [6]. Although the route and timing of the postulated arrival of the *Cx. pipiens* complex to the New World are controversial [8], if *Cx. quinquefasciatus *is a relatively recent arrival to the New World it may still retain the genetic signature of a founder event. 

 Our primary goal in the present study was to utilize mitochondrial DNA (mtDNA) sequences from a segment of the cytochrome *c* oxidase subunit I (COI) gene, known as the barcode segment [9], to examine population genetics of *Culex* mosquitoes collected from widely separated localities in the Sonoran Desert region, including the Baja California peninsula, mainland Sonora, Mexico and southeastern Arizona, USA, to test the prediction that *Cx. quinquefasciatus* and *Cx. tarsalis* will show evidence of different demographic histories. Because we found extremely low genetic variability in the COI gene in *Cx. quinquefasciatus*, we also used a small set of nuclear microsatellite loci to obtain preliminary estimates of population structure in this species. Also, because it is well known that taxonomic identifications based on morphological examination of females of certain species of *Culex *can be especially problematic [5, 10], COI barcodes, which can reliably distinguish many nominal species of *Culex* [11, 12], were used to estimate the overall biodiversity and phylogenetic relationships of *Culex* obtained in our survey.

## 2. Materials and Methods

### 2.1. Sampling

**Figure 1:**
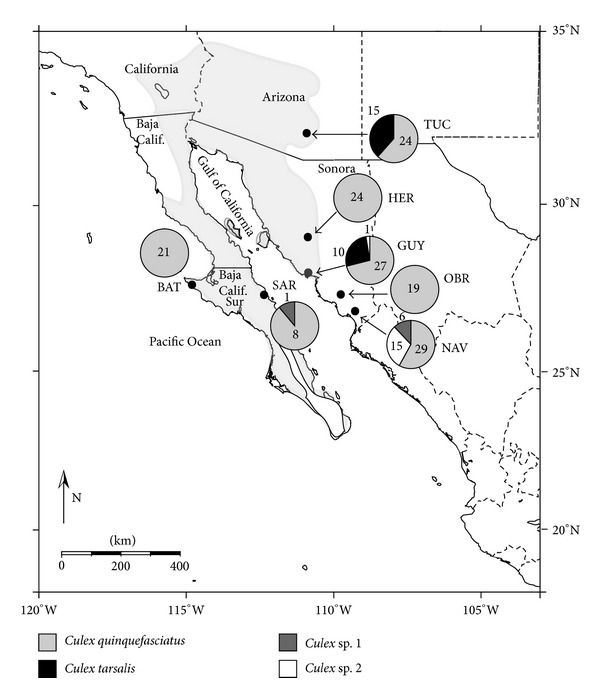
Map showing collection localities for *Culex* spp. in northwestern Mexico and southern Arizona, USA. Light gray shading on map shows approximate boundaries of the Sonoran Desert. Pie charts show numbers of individuals of each species analyzed from each locality. TUC: Tucson; HER: Hermosillo; GUY: Guaymas; OBR: Ciudad Obregón; NAV: Navojoa; SAR: Santa Rosalía; BAT: Bahía Tortugas.

Adult female mosquitoes were collected at seven localities, including the states of Sonora (Hermosillo, Guaymas, Ciudad Obregón, and Navojoa) and Baja California Sur (Bahía Tortugas and Santa Rosalía) in Mexico and southeastern Arizona (Tucson) in the USA (Figure 1). Ciudad Obregón and Navojoa are located in a thornscrub biome south of the Sonoran Desert, but are included here as part of the Sonoran Desert region. Carbon dioxide traps (BioQuip, Rancho Dominguez, CA, USA) were placed at collection sites from 1700 until 0900 when mosquitoes were collected from the traps. Mosquitoes were provisionally identified by examination under a dissecting microscope and then either frozen in liquid nitrogen, placed on dry ice, or immediately used for DNA extractions.

### 2.2. Molecular Protocol and Sequence Analysis

Total genomic DNA was extracted from each mosquito using the DNeasy (QIAGEN Inc., Valencia, CA, USA) protocol. Samples not analyzed immediately were stored at −20°C. The polymerase chain reaction (PCR) was used to amplify a segment of the COI gene using the primer pair LCO1490f/HCO2198r and standard assay conditions [13]. Sequencing reactions were performed on an Applied Biosystems (Foster City, CA, USA) ABI 3730XL DNA sequencer at the Genomic Analysis and Technology Core Facility, University of Arizona, Tucson, USA, using the amplifying primers. Sequences were proofread and aligned in either Sequencher 4.1 (GeneCodes Corp., Ann Arbor, MI, USA) or ClustalX 1.81 [14] followed by manual editing. Sequences were trimmed to remove ambiguous sites, resulting in a final segment of 624 bp in 23 of the 25 *Cx. tarsalis* (see Table 1) and 611 bp in *Cx. quinquefasciatus* and *Culex* sp. 1 and sp. 2. The first nucleotide in the 624 bp segment of *Cx. tarsalis* corresponds to position no. 1527 in the complete mitochondrial genome of *Drosophila yakuba *(GenBank Accession no. NC001322). The first nucleotide position in *Cx. quinquefasciatus* and *Culex *sp. 1 and sp. 2 corresponds to position no. 1515 in *D. yakuba*. GenBank accession numbers for the new *Culex* COI sequences obtained here are JX297260–JX297304.

With one exception, all individuals of *Cx. quinquefasciatus* possessed the same COI haplotype. To obtain a preliminary estimate of population structure in *Cx. quinquefasciatus*, therefore, we also analyzed four microsatellite loci (CQ16, CQ26, CQ29, and CQ41) as described by Fonseca et al. [15]. Most of the 134 specimens of *Cx. quinquefasciatus* analyzed for microsatellites were not the same as those used for COI analyses. Several individuals from Hermosillo (*N* = 6), Guaymas (*N* = 2), and Santa Rosalía (*N* = 2), however, were analyzed for both molecular markers. Genetic diversity for each locus in each of the seven populations (Figure 1), as well as over all loci and populations, was quantified using Microsatellite Analyser (MSA) version 4.00 [16] and ARLEQUIN version 3.5.1.3 [17]. Deviations from Hardy-Weinberg equilibrium (HWE) were tested for each locus and over all loci in ARLEQUIN using a Markov chain approximation [18]. All estimates were assessed for significance using a test analogous to Fisher's exact test, with 100,000 steps in the Markov chain and 5000 dememorization steps. Significance for all estimates was placed at the 0.05 level. Other details on the microsatellite protocol are given elsewhere [19]. 

 Calculations of Kimura's [20] 2-parameter genetic distances (*d*) were carried out in MEGA version 5.0.5 [21]. Genetic diversity indices were calculated in DnaSP version 5.00.07 [22]. Neutrality tests (Tajima's *D* [23] and Fu's *F*_*S*_ [24]) were carried out in ARLEQUIN. Fu's *F*_*S*_ test is also useful for detecting signatures of population expansions, which lead to large negative values in the test statistic [24, 25]. The significance of *F*_*S*_ at the 0.05 level is indicated when *P* values are <0.02 [17]. Networks for COI haplotypes were constructed using statistical parsimony implemented in TCS version 1.21 [26]. The connection limit among haplotypes was set to the default value of 95%, unless indicated otherwise.

### 2.3. Phylogenetic Analyses

Relationships among COI haplotypes in Sonoran Desert *Culex* were examined using maximum parsimony (MP) and Bayesian inference. For all phylogenetic analyses, sequences for *Cx. tarsalis* were trimmed from 624 to 611 bp to correspond to the sequence length of the other samples (Table 1). We also incorporated GenBank sequences for several different species of *Culex* into the data matrix, including *Cx. *(*Neoculex*) *territans* Walker and *Cx*. (*Culiciomyia*) *nigropunctatus* Edwards. All other *Culex* species treated here are presently assigned to the subgenus *Culex* [4]. *Culiseta inornata* (Williston) from the tribe Culisetini was used as the outgroup based on results of previous molecular studies of Culicidae [11, 27]. Maximum parsimony analyses were carried out in MEGA using the CNI heuristic search option and 100 random additions of sequences. Relative support for tree topology was obtained by bootstrapping [28] using 1000 pseudoreplicates. Bayesian analyses were implemented in MrBayes version 3.1 [29]. The model of nucleotide substitution that best fitted the data set, determined with jModelTest 0.1.1 [30] using the Akaike Information Criterion was, TVM + G. The substitution model was set to nst = “2” and rates = “gamma”, and the analysis was run for 1,000,000 generations, sampled every 250th generation (4,000 trees sampled), using the default random tree option to begin the analysis. We also conducted an analysis with nst = “6,” used for the more highly parameterized GTR substitution model, and obtained the same tree topology and similar clade support values. Clade support, expressed as posterior probabilities, was estimated utilizing a Markov chain Monte Carlo (MCMC) algorithm.

### 2.4. Population Structure and Historical Demography

Analysis of molecular variance (AMOVA) [31], performed in ARLEQUIN, was used to test for population structure among populations of *Cx. quinquefasciatus* and *Cx. tarsalis*. The significance of population pairwise comparisons of the fixation indices, Φ_ST_ for COI and *F*_ST_ for microsatellites, was based on 10,000 permutations of the data matrix and assessed at *α* = 0.05 (*Cx. tarsalis*) or using a sequential Bonferroni correction [32] for multiple comparisons of *Cx. quinquefasciatus*. Estimates of the number of migrants per generation (*N*_*m*_) among populations were also calculated in ARLEQUIN.

 The demographic history of *Cx. tarsalis* from the Sonoran Desert was inferred by performing three different tests of the sequence data. For all demographic tests, we chose a value of 2.3% pairwise sequence divergence per million years for COI [33]. This resulted in a neutral mutation rate per site per generation (*μ*) of 1.15 × 10^−8^ assuming a single generation per year (see Section 4). A mismatch distribution analysis [34, 35] of COI sequence data was performed in ARLEQUIN. The significance of the estimated parameters of the sudden expansion model of the mismatch distribution is obtained from the sum of square deviations (SSD) statistic and the raggedness statistic (rg) and their corresponding *P* values. The sudden expansion model is rejected at *P* < 0.05. A Bayesian skyline analysis, which provides an estimate of changes in effective population size through time utilizing MCMC sampling of sequence data, was conducted in BEAST version 1.3 [36]. Because the TVM substitution model is not available in BEAST, analyses were run using both the HKY + G and GTR + G substitution models (four gamma categories) for five million iterations sampled every 1000 iterations. Bayesian skyline plots generated with TRACER version 1.5 [36] were essentially identical in the two analyses. A maximum-likelihood estimate of the exponential population growth parameter (*g*) and the mutation parameter *θ* in *Cx. tarsalis* was obtained with the program FLUCTUATE version 1.4 [37] using the program settings described previously [38].

## 3. Results

### 3.1. Sequence Analysis

*Culex* COI sequences were translated in MEGA. No frameshifts or stop codons were found. Base composition showed little variation among sequences, with CG content averaging 31%. Together these results suggest that our sequences represent mtDNA and are not nuclear mitochondrial pseudogenes (numts) which have been reported for the COI gene in insects [39]. 

 Genetic diversity indices and results of neutrality tests for COI are shown in Table 1. The very low haplotype (*h*) and nucleotide (*π*) diversities found in *Cx. quinquefasciatus* contrast markedly with the high values seen in *Cx. tarsalis* and the two unidentified species. Tajima's *D* was not significant in any of the *Culex *species. A relatively large and significant Fu's *F*_*S*_, however, was found in *Cx. tarsalis*.

### 3.2. Phylogenetic Relationships

**Figure 2:**
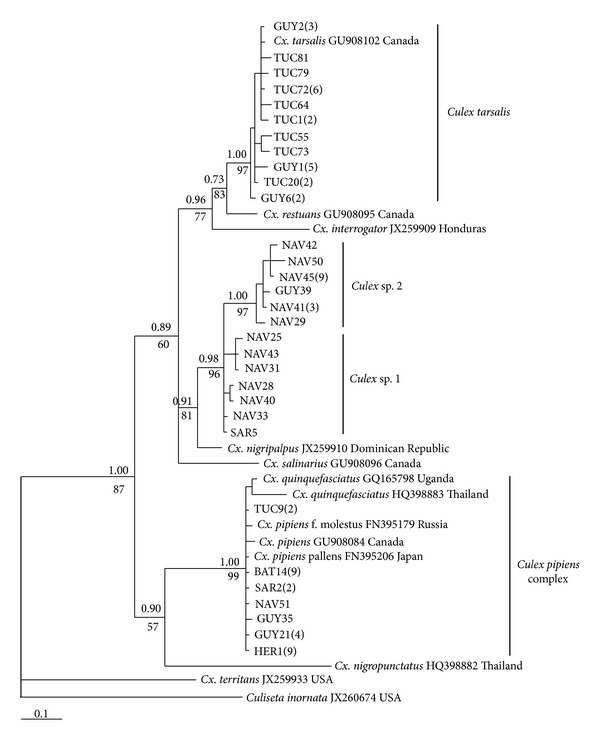
Bayesian 50% majority rule consensus tree based on COI sequences (611 bp) showing relationships among haplotypes of *Culex* spp. from the Sonoran Desert region and GenBank sequences from several recognized species worldwide. Clade support expressed as posterior probabilities is shown above the branches. Bootstrap support values for the maximum parsimony (MP) tree (length = 235; CI = 0.698; RI = 0.677; 154 variable sites; 90 parsimony informative sites) are shown below the branches. Branch terminals are labeled with locality abbreviation and sample identification number (see Figure 1). The total number of identical haplotypes from each region is shown in parentheses following the listed haplotype. The scale shows substitutions per site.

Phylogenetic relationships among COI haplotypes from 76 individuals of *Culex* collected from six Sonoran Desert localities (mosquitoes from Ciudad Obregón were analyzed only for microsatellites) revealed four well-resolved clades (Figure 2). One clade found at all six localities (*N* = 28) clustered with the closely related *Cx. pipiens* and *Cx. quinquefasciatus* (*Cx. pipiens* complex). Of these 28 individuals, 27 possessed the same haplotype, which was identical to the corresponding 611 bp COI segment reported for *Cx. pipiens pallens* from Japan (GenBank Accession no. FN395206). Another clade found only at Tucson and Guaymas comprised *Cx. tarsalis* (*N* = 25). The remaining two clades, found mainly at Navojoa and provisionally assigned to *Culex* sp. 1 and sp. 2 (Figure 1), did not cluster with any of the available GenBank sequences and remained unidentified (see Section 4). *Culex nigripalpus* from the Dominican Republic (GenBank Accession no. JX259910), however, resolved in a basal position to the two unidentified *Culex* and was closely related to them (*d* = 2.2 − 3.9%), supporting their assignment to the genus *Culex*. Mean genetic distance between *Culex* sp. 1 and sp. 2 was *d* = 2.1%. *Culex *sp. 1 and sp. 2 also differed from all other species of *Culex* analyzed here by showing a nonsynonymous first codon nucleotide substitution (G to A) at position 68 of the COI gene segment which resulted in an alanine to threonine substitution in the COI protein.

**Figure 3:**
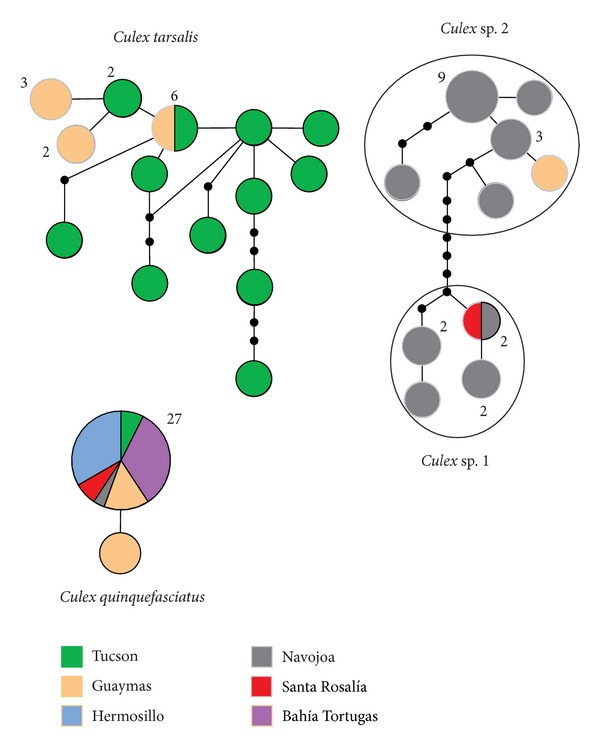
TCS haplotype network for the COI gene segment in *Culex tarsalis* (*N* = 23; see Table 1), *Cx*. *quinquefasciatus* (*N* = 28), *Culex* sp. 1 (*N* = 7), and *Culex* sp. 2 (*N* = 16) based on a 95% connection limit. Each line segment represents a single mutation. Inferred intermediate haplotypes that were not sampled are shown as small black dots on the line segments. Size of the circles is proportional to haplotype frequency. Numbers next to the circles represent number of individuals with that haplotype, if greater than one. Geographical localities are color-coded.

The TCS analyses showed that COI haplotypes for *Cx. tarsalis* and the two unidentified *Culex* species resolved in separate networks at the 95% connection limit (Figure 3), consistent with the presence of at least two species-level taxa. The clustering of haplotypes for *Culex* sp. 1 and sp. 2 in a network separate from *Cx. tarsalis* indicates that the unidentified species are closely related, although separated by nine mutational steps. When the connection limit was increased from 95 to 97% in the TCS analysis, *Culex *sp. 1 and sp. 2 formed separate networks (not shown) supporting the view that they represent separate species.

Of the fourteen haplotypes seen in *Cx. tarsalis* (Table 1), ten were singletons, all of which were found at Tucson (Figure 3). The abundance of singleton haplotypes suggests an expanding population, consistent with results from the demographic tests (see Section 3.4). Although the common haplotype in *Cx. tarsalis* was present at both Guaymas and Tucson, the geographic partitioning shown in Figure 3 also is consistent with the results from the AMOVA showing significant structure between Guaymas and Tucson populations (see Section 3.3). 

### 3.3. Population Structure

A summary of results obtained for the four microsatellite loci in *Cx. quinquefasciatus* averaged over the seven Sonoran Desert populations is shown in Table 2. All four loci showed significant deviations (*P* < 0.01) from HWE. In all cases we found *H*_obs_< *H*_exp⁡_ indicating an excess of homozygotes. When each population was analyzed separately, however, no significant deviations from HWE were found at the CQ26 locus, and most populations showed no significant deviations at the CQ16 and CQ29 loci (not shown). For the CQ41 locus, HWE was found only in populations at Ciudad Obregón, Santa Rosalía, and Tucson. An excess of homozygotes has also been reported in other microsatellite studies of the *Cx. pipiens* complex and has been attributed to the presence of null alleles [40] or the Wahlund effect [7].

AMOVA of the microsatellite data set for *Cx. quinquefasciatus* from seven localities revealed that only five of the 21 pairwise comparisons of *F*_ST_ were significant after Bonferroni correction for multiple comparisons (Table 3). 

The AMOVA of the COI data set in *Cx. tarsalis *collected from Tucson (*N* = 15) and Guaymas (*N* = 8) showed that 85% of the genetic variation was found within populations, but significant structure was found between populations from the two localities (Φ_ST_ = 0.15; *P* = 0.007). The estimated number of migrants per generation (*N*_*m*_) between Tucson and Guaymas, however, was 2.8. Therefore, for demographic analyses (Section 3.4), the two populations were combined.

### 3.4. Historical Demography

FLUCTUATE showed that the population growth parameter (*g* ± 95% confidence interval) in *Cx. tarsalis*, expressed in units of 1/*μ* generations, was positive and significantly different from zero (*g* = 664 ± 165), consistent with population growth. The maximum-likelihood estimate for the mutation parameter *θ* was 0.027363 ± 0.00773. Effective female population size (*N*_ef_) in *Cx. tarsalis*, estimated using the equation *θ* = 2*N*_ef_ *μ*, was 1.19 × 10^6^.

**Figure 4:**
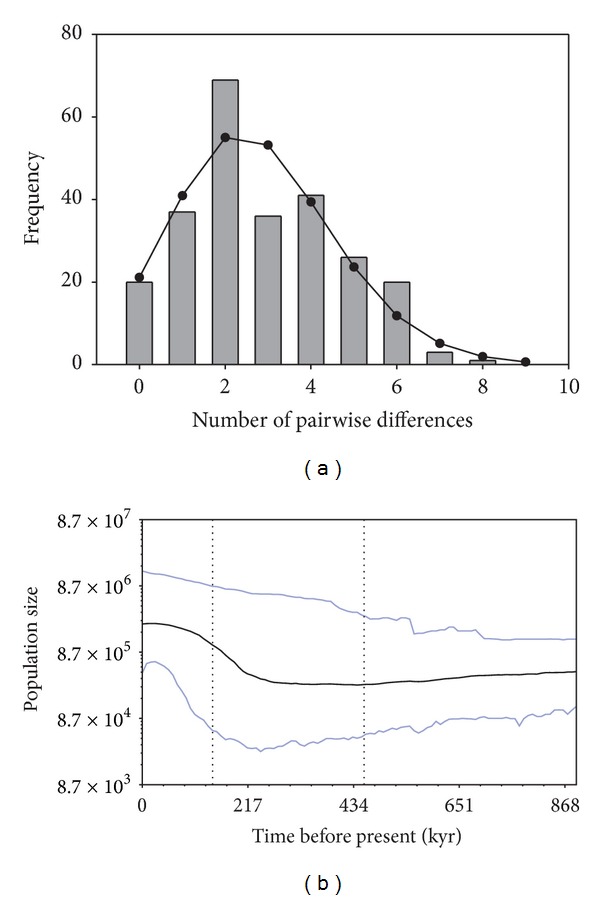
Demographic history of *Culex tarsalis* from the Sonoran Desert inferred from the mismatch distribution (a) and Bayesian skyline analysis (b). Vertical bars of the mismatch distribution show the observed distribution of pairwise differences among COI haplotypes, with the solid line representing the expected distribution under the sudden expansion model. The Bayesian skyline plot shows the estimated changes in effective female population size (*N*_ef_) over time given on a logarithmic scale. The solid lines represent the median estimates of population size (middle line); upper and lower lines show the 95% highest posterior density (HPD) intervals. The vertical dotted lines represent the median estimate (right) and lower 95% HPD (left) of time to the most recent common ancestor.

 The mismatch distribution of COI sequences in *Cx. tarsalis* is shown in Figure 4. The observed distribution of pairwise differences among haplotypes showed relatively good agreement with the expected unimodal distribution for a population that has undergone an expansion [35]. The test statistics SSD (0.0092; *P* = 0.37) and rg (0.047; *P* = 0.44) were small and not statistically significant, indicating that the sudden expansion model could not be rejected. The value found for *τ*, the time to the population expansion, where *τ* = 2*ut*, *u* is the mutation rate for the entire gene segment, and *t* is the number of generations since the expansion [34], was 3.029 (95% confidence intervals: 1.023, 4.896). Assuming 2.3% pairwise divergence per million years in the COI gene in insects [33], the mean mutation rate per site per generation in the 624 bp segment for a single lineage is (624) × (1.15 × 10^−8^) or 7.176 × 10^−6^. Based on these values, the estimated time to the population expansion in *Cx. tarsalis* (with 95% confidence intervals) was 211,050 (71,279–341,140) generations ago.

 Bayesian skyline analysis (Figure 4) showed that *Cx. tarsalis* showed a clear signature of an historical population expansion, consistent with the results from FLUCTUATE and the mismatch distribution. Given the untested assumptions of a neutral mutation rate per site per generation (*μ*) of 1.15 × 10^−8^ and a single generation per year, the timing of the expansion shown in the Bayesian skyline plot is only a rough approximation. Nonetheless, the mismatch distribution and Bayesian skyline plot both suggest that the expansion began approximately 200,000 generations ago, which places it within the timeframe of the Pleistocene, unless improbable estimates of *μ* and generation time are assumed.

## 4. Discussion

### 4.1. Genetic Diversity

A major finding of this study was that genetic diversity in the COI gene segment of *Cx. quinquefasciatus* from the Sonoran Desert was much lower than that seen in *Cx. tarsalis* and *Culex* sp. 1 and sp. 2 (Table 1). One possible explanation for this difference is that *Cx. quinquefasciatus* has preferentially undergone repeated cycles of population fluctuations, resulting in a much lower genetic diversity, owing to vector control measures in urban areas in northwestern Mexico which are primarily aimed at controlling *Ae. aegypti* and the dengue virus. Subtle ecological differences in microhabitat preferences that result in less exposure to insecticides might explain why the Sonoran Desert *Cx. tarsalis* and *Culex* sp. 1 and sp. 2 maintain a relatively high genetic diversity. These three species, or putative species, show diversity indices similar to native dipterans from the Sonoran Desert region, including the cactophilic *Drosophila* (with the exception of *D. nigrospiracula*) and *Odontoloxozus longicornis* and *O. pachycericola* [38, 41–43]. Ecological studies conducted in California, USA, have shown that both *Cx. quinquefasciatus* and *Cx. tarsalis* are most abundant in riparian habitats, but that *Cx. quinquefasciatus* shows higher relative abundance than *Cx. tarsalis* in residential habitats [44]. We also noted that *Cx. quinquefasciatus* was typically the most abundant mosquito species in our urban collections, consistent with the observations of Reisen et al. [44]. 

Another possible explanation for the large differences in genetic diversity among Sonoran Desert *Culex*., which is not mutually exclusive of the above hypothesis, may be related to differences in population histories. *Culex tarsalis* is native to North America, whereas both *Cx. quinquefasciatus* and *Cx. pipiens* probably evolved in Africa [6]. Ross [45] hypothesized that *Cx. quinquefasciatus* was introduced from Africa via the slave trade within the last few centuries. This hypothesis, however, has been challenged [5]. Regardless of the dispersal route, if *Cx. quinquefasciatus* is a relatively recent arrival to the New World [8], it is possible that it would still retain the genetic signature of a founder event (i.e., reduced genetic variability) compared with the indigenous *Cx. tarsalis*.

A recent study reported the presence of *Cx. pipiens*, and hybrids between *Cx. pipiens* and *Cx. quinquefasciatus*, in Mexico City [46]. Given the close association of both species with humans, together with their potential for dispersal via commercial air traffic, clear patterns of global distributions of the two species and their hybrids [5] may become progressively obscured. Figure 2 shows that COI barcode sequences are unable to distinguish between the two species. Although a possibility thus exists that our samples are *Cx. pipiens*, or hybrids of *Cx. pipiens* and *Cx. quinquefasciatus*, a more thorough examination of individuals of the *Cx. pipiens* complex from this region with specific molecular markers that reliably separate the two cryptic species [5, 8, 10] will be required before this can be resolved.

Females of several species of *Culex* from North America, including *Cx. restuans* Theobald, *Cx. nigripalpus* Theobald, and *Cx. salinarius* Coquillett, are often indistinguishable from those of the *Cx. pipiens* complex and can easily be confused [5, 10]. Barcode sequences, however, have been shown to be useful for separating and identifying species of Culicidae, with interspecific K2P divergences of COI generally showing values ≥2% [11, 12, 47]. Based on the 2% cutoff, we provisionally assigned the unidentified lineages to *Culex* sp. 1 and sp. 2 (mean *d* = 2.1%), but further molecular and morphological studies will be required to confirm their identity. We have shown, however, that *Culex* sp. 1 and sp. 2, which were initially identified as *Cx. quinquefasciatus* based on morphological examination, are very closely related to *Cx. nigripalpus* from the Dominican Republic (Figure 2) and only more distantly related to the other *Culex* species shown in Figure 2. When we compared sequences of *Culex* sp. 1 and sp. 2 with shorter COI barcode segments (478 bp) of *Culex* from Brazil [48], they also did not cluster with *Cx. bidens* Dyar, *Cx. corniger* Theobald, *Cx. coronator* Dyar & Knab, or *Cx. nigripalpus*, species that have been recorded for the states of Sonora and/or Sinaloa [49].

### 4.2. Population Structure

Field studies suggest that both *Cx. quinquefasciatus* and *Cx. tarsalis* show relatively high dispersal capability [44]. The AMOVA results of preliminary microsatellite data on *Cx. quinquefasciatus* from seven widely separated Sonoran Desert localities, including localities separated by the Gulf of California (Figure 1), showed that most of the pairwise comparisons of *F*_ST_ were not significant, consistent with a pattern of high gene flow. None of the pairwise comparisons of *F*_ST_ between the peninsular locality at Santa Rosalía and five mainland localities were significant. The most isolated locality at Bahía Tortugas on the Pacific coast of the peninsula also showed a lack of structure between both Guaymas and Hermosillo, although the pairwise comparisons with the other three mainland localities were significant. These results are in contrast to the findings from microsatellite studies on the closely related *Cx. pipiens* in Colorado in which significant structure was found among populations within the state [7]. Our results, however, are similar to those obtained from microsatellite studies on *Cx. pipiens* populations from several states in northeastern USA in which most pairwise comparisons among populations were not significant [40]. Our findings are also consistent with results from several species of native cactophilic dipterans which show little or no structure within mainland and peninsular populations, and, in the case of *Drosophila nigrospiracula* and *D. mettleri*, no apparent structure between peninsular and mainland populations [50].

 Because we were unable to distinguish *Culex* sp. 1 and sp. 2 from *Cx. quinquefasciatus* using morphological characters, we cannot rule out the possibility that individuals of these two unidentified putative species were present in our sample assigned to *Cx. quinquefasciatus* from Navojoa used for microsatellite analysis. None of the Navojoa DNA samples identified as *Cx. quinquefasciatus* and analyzed for microsatellites were sequenced for COI to confirm their identity. As mentioned earlier, DNA extracted from six individuals of *Cx. quinquefasciatus *from Hermosillo were analyzed for both molecular markers. The observation that no significant population structure was found between *Cx. quinquefasciatus* from Hermosillo and each of the other populations (Table 3), and the observation that no individuals of *Culex* sp. 1 and sp. 2 were found at Hermosillo, suggests that few, if any, of the individuals in our microsatellite sample from Navojoa contained *Culex* sp. 1 and sp. 2.

 Previous studies have examined the population genetic structure of *Cx. tarsalis *in western USA utilizing microsatellite markers [51, 52]. These studies have revealed a pattern of little population structure across broad areas at both the state (Colorado) and regional levels, consistent with high dispersal capability, although evidence of restriction of gene flow related to major geographic barriers (e.g., Continental Divide, Mogollon Rim, and the transition between Sonoran and Mojave Deserts) was evident. In particular, Venkatesan and Rasgon [52] found three separate population clusters of *Cx. tarsalis* in western USA. One of these clusters, the Sonoran cluster, occurred in southern Arizona and southeastern California, and included our sampling site at Tucson. Although we found significant structure between Guaymas and Tucson, a distance of approximately 500 km, the estimated number of migrants per generation among the two localities (*N*_*m*_ = 2.8) suggests some gene flow. Larger sample sizes of *Cx. tarsalis* from the Sonoran Desert are needed, but our preliminary results based on COI are consistent with the microsatellite data in suggesting that some restrictions to gene flow may also occur in this region. 

### 4.3. Demographic History

The large and significant negative value for Fu's *F*_*S*_ seen in *Cx. tarsalis* from the Sonoran Desert (Table 1) suggested an historical population expansion. This conclusion was supported by results from FLUCTUATE, the mismatch distribution, and Bayesian skyline analysis. The mismatch distribution indicated that the population expansion began approximately 211,000 generations ago. Given the large confidence intervals surrounding the estimated number of generations since the expansion obtained from the mismatch distribution, and uncertainties in the generation time for *Cx. tarsalis* from the Sonoran Desert, it is impossible to arrive at a specific date for the expansion. A conservative estimate of 5–10 generations per year, however, would place the expansion at about 20,000–40,000 years ago during the late Pleistocene. This estimated timeframe is much more recent than that obtained by Venkatesan et al. [53] based on the ND4 gene in *Cx. tarsalis*, in which the expansion was dated to about 11,300,000 generations ago, or 375,000–560,000 years ago using their assumption of 20–30 generations per year. Although different genes, sample sizes, and assumptions were used in the two studies, a calculation error [54] is suspected in the ND4 study which probably contributed to the large discrepancy in estimated expansion dates.

## Figures and Tables

**Table 1:** Summary of genetic diversity indices and results of neutrality tests (Tajima's *D* and Fu's *F*_*S*_) in the mitochondrial COI gene segment of *Culex* spp.

Species	*N*	*L*	*k*	*K*	*h* (±SD)	*π* (±SD)	Tajima's *D*	Fu's *F*_*S*_
*Cx. quinquefasciatus *	28	611	1	2	0.071 ± 0.065	0.00012 ± 0.00011	−1.15	−1.15*
*Cx. tarsalis *	23**	624	15	14	0.921 ± 0.042	0.00460 ± 0.00069	−1.05	−7.54*
*Culex* sp. 1	7	611	5	4	0.857 ± 0.102	0.00405 ± 0.00075	1.06	0.28
*Culex* sp. 2	16	611	8	6	0.675 ± 0.117	0.00218 ± 0.00066	−1.61	–1.81

*N*: number of sequences; *L*: sequence length (bp); *k*: number of variable sites; *K*: number of haplotypes; *h*: haplotype diversity; *π*: nucleotide diversity; *significant at the 0.05 level; **two of the 25 sequences obtained for *Cx. tarsalis* (GUY1 and GUY3) contained 558 bp and were omitted.

**Table 2:** Summary information of the four microsatellite loci averaged over the seven populations of *Culex quinquefasciatus*. The number of individuals genotyped (*N*), observed and expected heterozygosities (*H*_obs_ and *H*_exp⁡_), fragment size range (bp), and number of alleles are shown for each locus.

Locus	*N*	*H*_obs_	*H*_exp⁡_	Size (bp)	No. of alleles
*CQ16 *	101	0.77228*	0.86813	210–260	17
*CQ26 *	132	0.62879*	0.68879	208–220	7
*CQ29 *	131	0.30534*	0.41628	168–180	5
*CQ41 *	133	0.51128*	0.68035	136–154	8

*Significant deviation (*P* < 0.01) from Hardy-Weinberg equilibrium (HWE).

**Table 3:** Pairwise comparisons of *F*_ST_ for populations of *Culex quinquefasciatus* from the Sonoran Desert region based on analyses of four microsatellite loci. Sample sizes from each locality are shown in parentheses. Locality abbreviations are given in the legend of Figure 1.

	TUC	HER	GUY	OBR	NAV	SAR	BAT
	(22)	(21)	(24)	(19)	(28)	(8)	(12)
TUC	—						
HER	0.007	—					
GUY	0.042*	−0.000	—				
OBR	0.006	0.011	0.074*	—			
NAV	0.029	−0.003	0.022	0.015	—		
SAR	0.019	−0.015	−0.059	0.017	−0.033	—	
BAT	0.068*	0.064	0.047	0.126*	0.099*	0.017	—

*Statistically significant values using a Bonferroni correction (*P* < 0.003).
